# The Effect of Solution Treatment Duration on the Microstructural and Mechanical Properties of a Cold-Deformed-by-Rolling Ti-Nb-Zr-Ta-Sn-Fe Alloy

**DOI:** 10.3390/ma17040864

**Published:** 2024-02-13

**Authors:** Vasile Dănuț Cojocaru, Nicolae Șerban, Elisabeta Mirela Cojocaru, Nicoleta Zărnescu-Ivan, Bogdan Mihai Gălbinașu

**Affiliations:** 1 Faculty of Materials Science and Engineering, National University of Science and Technology Politehnica of Bucharest, 060042 Bucharest, Romania; dan.cojocaru@upb.ro (V.D.C.); nicolae.serban@upb.ro (N.Ș.); elisabeta.cojocaru@upb.ro (E.M.C.); 2 Dental Medicine Faculty, University of Medicine and Pharmacy “Carol Davila” Bucharest, 020021 Bucharest, Romania; bogdan.galbinasu@umfcd.ro

**Keywords:** β-Ti phase, cold-rolling deformation, solution treatment, SEM analysis, X-ray diffraction analysis, microstructural characteristics, mechanical properties

## Abstract

The study presented in this paper is focused on the effect of varying the solution treatment duration on both the microstructural and mechanical properties of a cold-deformed by rolling Ti-30Nb-12Zr-5Ta-2Sn-1.25Fe (wt.%) alloy, referred to as TNZTSF. Cold-crucible induction using the levitation synthesis technique, conducted under an argon-controlled atmosphere, was employed to fabricate the TNZTSF alloy. After synthesis, the alloy underwent cold deformation by rolling, reaching a total deformation degree (total applied thickness reduction) of 60%. Subsequently, a solution treatment was conducted at 850 °C, with varying treatment durations ranging from 2 to 30 min in 2 min increments. X-ray diffraction (XRD) and scanning electron microscopy (SEM) techniques were utilized for the structural analysis, while the mechanical properties were assessed using both tensile and hardness testing. The findings indicate that (i) in both the cold-deformed-by-rolling and solution-treated states, the TNZTSF alloy exhibits a microstructure consisting of a single β-Ti phase; (ii) in the solution-treated state, the microstructure reveals a rise in the average grain size and a decline in the internal average microstrain as the duration of the solution treatment increases; and (iii) owing to the β-phase stability, a favorable mix of elevated strength and considerable ductility properties can be achieved.

## 1. Introduction

Compared to other metallic implant materials used for orthopedic applications, like medical stainless steel (316L SS) or Co-Cr alloys, it has been proven in many studies that Titanium (Ti) and Ti alloys possess a more outstanding performance due to their high strength, high fracture toughness, good corrosion resistance, relatively low elastic modulus, and good biocompatibility [1,2,3,4,5,6,7].

To date, commercially pure Ti and Ti-6Al-4V (Ti64) are the most widely used for Ti-based orthopedic implants [8,9,10,11,12]. Although Ti64 exhibits an elastic modulus (E ≈ 100–110 GPa) [9,11] twice lower than medical stainless steel (E ≈ 200–210 GPa) [13,14] or Co–Cr alloys (E ≈ 220–230 GPa) [15,16], this value is about four times bigger than that of human bone (E ≈ 10–30 GPa) [17,18]. It seems that the main reason why Ti64 alloys have shown tendencies to fail after long-term use is that their elastic modulus values are higher than that of human bone, thereby causing the “stress shielding effect” [19,20]. Since Aluminum (Al) and Vanadium (V) have been reported to have toxic effects on the human body, the lack of biocompatibility could be another reason for failure after the long-term use of Ti-6Al-4V alloys [21,22].

It is well known that the two main factors that influence the biocompatibility of a metallic material used as an implant are the response of the human body induced by the material and the degradation of the material in the human environment. Therefore, in recent years, a lot of efforts have been made regarding the development of Ti-based implants having an elastic modulus comparable to that of human bone and non-toxicity, as well as good resistance inside the human body. Although many studies have been carried out, there is currently no Ti-based alloy that simultaneously presents all the requirements of an implant for long-term use, like a low elastic modulus, good wear resistance, biocompatibility, and excellent corrosion resistance.

Recent works have focused on β-Ti alloys containing non-toxic β-stabilizing elements like Niobium (Nb), Zirconium (Zr), Tantalum (Ta), Tin (Sn), and Iron (Fe), which generally possess good mechanical properties, excellent corrosion resistance, low elastic moduli, and superior biocompatibility [23,24,25,26,27]. In Ti-based alloys, β-stabilizing elements, like Nb and Ta, are usually added to obtain the β phase at room temperature, and Zr and Sn are added to achieve better β-phase stability, thus enhancing its strength, while Fe, being a strong β-stabilizing element, is added to replace some expensive β-stabilizing elements (Nb and Ta) and to obtain a visible solid-solution strengthening effect at the same time [28,29,30].

To improve the mechanical efficiency of Ti-based alloys used as implants, thermo-mechanical processing (TMP) is considered to be effective by many researchers [31,32,33,34,35]. Thermo-mechanical processing consists of two main stages. In the first stage, a Ti-based alloy is cold-deformed by rolling (CR), which can achieve grain refinement and strain-hardening effects, thus improving its strength, and in the second stage, the CR Ti-based alloy is solution-treated (ST) at different temperatures, whereby the strength of the alloy can be further enhanced due to the precipitation strengthening effect. So, by using a suitable thermo-mechanical processing route by controlling the cold-rolling deformation degree and heat treatment conditions, Ti-based alloys with improved mechanical properties can be obtained.

Research has been expanded in recent years in an attempt to find the most efficient thermo-mechanical processing route (TMP) that enhances β-Titanium alloys’ microstructural and mechanical properties. A distinctive thermo-mechanical processing approach was employed in this study. Therefore, in this study, a Ti-30Nb-12Zr-5Ta-2Sn-1.25Fe (TNZTSF) (wt.%) alloy was subjected to a 60% cold-rolling deformation degree (total applied thickness reduction), followed by a solution treatment at 850 °C with variable treatment durations from 2 min to 30 min in 2 min increments, which achieved a good combination of high strength and a low elastic modulus. The elastic modulus of the TNZTSF alloy was expected to decrease due to the stress-induced phase transformation that takes place during cold-rolling deformation, and the mechanical properties of the TNZTSF alloy were expected to be improved by the precipitates generated during the solution treatment.

This research aimed to study the influence of the solution treatment duration on the microstructural and mechanical properties of a cold-rolled TNZTSF alloy and to develop a microstructure that has a good balance between the elasticity modulus and high strength using the proper thermo-mechanical processing route. Therefore, a detailed investigation of the microstructure evolution, mechanical properties, and deformation mechanisms was carried out.

## 2. Materials and Methods

### 2.1. Manufacture of the Alloy

The TNZTSF alloy examined in this study is made up of only high-purity elements, including Ti (minimum 99.6%), Nb (minimum 99.9%), Zr (minimum 99.5%), Ta (minimum 99.9%), Sn (minimum 99.96%), and Fe (minimum 99.98%). It was produced in an inert controlled atmosphere (argon) using a FIVE CELES-MP25 cold-crucible induction levitation furnace (Five’s Group Company, Paris, France).

The acquired ingots were subjected to three rounds of remelting to guarantee a high level of chemical homogeneity in the alloy. The chemical composition of the alloy was analyzed using the Energy-Dispersive X-ray Spectroscopy (EDS) technique, employing a TESCAN VEGA II XMU scanning electron microscope (SEM) (TESCAN, Brno, Czech Republic) in conjunction with a BRUKER Quantax xFlash 6/30 EDS detector (Bruker Corporation, Billerica, MA, USA).

### 2.2. Thermo-Mechanical Processing of the Alloy

**Figure 1:**
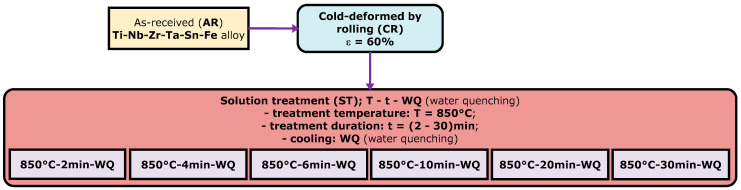
The thermo-mechanical processing (TMP) route implemented for the TNZTSF alloy.

In Figure 1, the employed thermo-mechanical processing route (TMP) is illustrated. As can be seen, initially, the as-received (AR) TNZTSF alloy went through a mechanical processing stage, namely, cold deformation by rolling (CR). CR was performed by using a Mario di Maio LQR120AS rolling mill (Mario di Maio Inc., Milano, Italy), achieving an entire deformation degree (thickness reduction) of approximately ε ≈ 60% in six uniform steps.

Following cold deformation by rolling, the TNZTSF samples underwent a thermal processing stage involving a solution treatment (ST) at 850 °C, in which the treatment duration was systematically adjusted within the range of 2 to 30 min. The solution treatment was conducted by using a CARBOLITE-GERO SR 100 × 500 furnace (Carbolite-Gero Inc., Neuhausen, Germany) operating under high-vacuum conditions. Finally, all the solution treatments were succeeded by water quenching (WQ).

### 2.3. Microstructural Analysis of the Alloy

**Figure 2:**
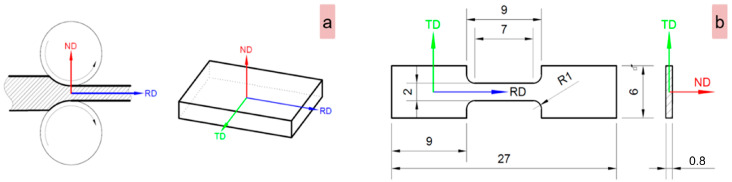
Sample analysis reference plane (RD-TD) (**a**) and schematic representation of the samples employed in tensile testing (**b**).

The microstructures of the as-received (AR), cold-deformed-by-rolling (CR), and solution-treated (ST) conditions were analyzed to evaluate various microstructural characteristics, such as the phase structure, morphology, grain size, etc. As shown in Figure 2a, all microstructural analyses were conducted in the reference plane of the rolling-direction–transverse-direction (RD-TD).

A Metkon MICRACUT 202 high-precision cutting device (Metkon Instruments Inc., Bursa, Turkey) equipped with an NX-MET XDLM diamond cutting disk (NX-MET, Echirolles, France) was employed for the sample-cutting process at all stages. Following the cutting procedure, the samples were embedded in a conductive phenolic resin using a Buehler SimpliMet2 (Buehler Ltd., Lake Bluff, IL, USA) hot-mounting press. The samples, once mounted, underwent initial polishing using a Metkon Digiprep ACCURA (Metkon Instruments Inc., Bursa, Turkey) machine. In order to enhance the surface quality further, an additional super-polishing step was then executed using a Buehler VibroMet2 (Buehler Ltd., Lake Bluff, IL, USA) machine. A more detailed explanation of the polishing and super-polishing steps can be found in a previous paper [33]. 

For the microstructural analysis, X-ray diffraction (XRD) and scanning electron microscopy (SEM) techniques were utilized. The XRD analysis was conducted by using a RIGAKU MiniFlex600 (RIGAKU, Tokyo, Japan) benchtop diffractometer, and the SEM analysis was performed with a TESCAN VEGA II XMU (TESCAN, Brno, Czech Republic) SEM microscope equipped with a Bruker eFlash1000 EBSD detector (Bruker Corporation, Billerica, MA, USA).

### 2.4. Mechanical Characterization of the Alloy

The samples for each structural condition (as-received (AR), cold-deformed by rolling (CR), and solution-treated (ST)) underwent mechanical characterization through tensile and microhardness testing. The tensile testing was conducted using a DEBEN MicroTest-2000N testing machine (Deben, Woolpit, UK) with a strain rate of around 1 × 10^−4^ s^−1^, while microhardness testing was conducted by employing a SHIMADZU HMV-2 microhardness tester (Shimadzu Corporation, Kyoto, Japan) with an applied force of 100 gf and a dwell duration of 30 s. From the acquired stress–strain curves, the subsequent mechanical properties were calculated: elasticity modulus (E), ultimate strength (σ_UTS_), yield strength (σ_0.2_), and fracture/failure strain (ε_f_).

As can be seen in Figure 2b, where a schematic representation of the samples employed in all tensile testing is given, “dog-bone”-shaped specimens that are characterized by calibrated dimensions of 2 × 0.8 × 7 mm were utilized.

## 3. Results

### 3.1. The Characterization of the TNZTSF Alloy in Its As-Received (AR) State

Before being subjected to cold deformation by rolling, the TNZTSF alloy used in this work, in its as-received (AR) state, was microstructurally, chemically, and mechanically characterized. The alloy’s microstructural analysis was performed by using both a scanning electron microscope (SEM) and an X-ray diffractometer (XRD). The chemical composition was examined by using the SEM-EDS technique, while the mechanical property analysis was performed by using a tensile test and microhardness test.

**Figure 3:**
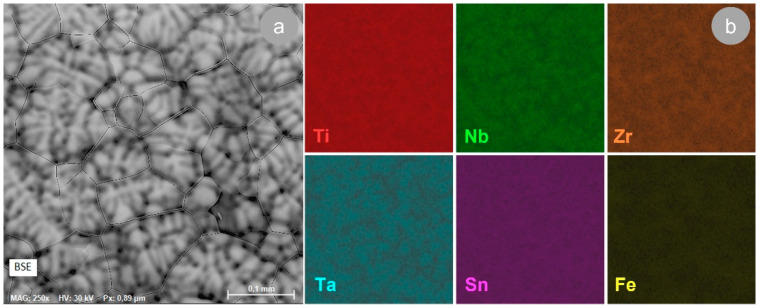
Microstructure of the TNZTSF alloy in the AR state (**a**); SEM-EDS colorized maps of the TNZTSF alloy in the AR state reflecting the primary alloying components’ distributions (**b**).

Figure 3a illustrates a demonstrative SEM image, achieved with a BSE (backscattered electron) detector, of the TNZTSF alloy in its AR state, which showcases its microstructure consisting of polyhedral grains, while Figure 3b illustrates the distribution of the primary alloying components in the structure of our AR TNZTSF alloy (Titanium, Niobium, Zirconium, Tantalum, Tin, and Iron) obtained by EDS elemental mapping.

By analyzing the EDS elemental maps, it can be noticed that (1) all alloying elements are evenly distributed, thus indicating that the alloy used in this study demonstrates a good chemical homogeneity, and (2) no other alloying components were found in the chemical composition of the TNZTSF alloy in its AR state. Therefore, it can be concluded that the production of the TNZTSF alloy through cold-crucible induction in the levitation synthesis technique, conducted in an argon-controlled atmosphere, was efficient, and a uniform chemical composition was obtained without any contamination. This can also be proven by the quantitative chemical composition analysis of the TNZTSF alloy in its AR state, which is summarized in Table 1.

**Figure 4:**
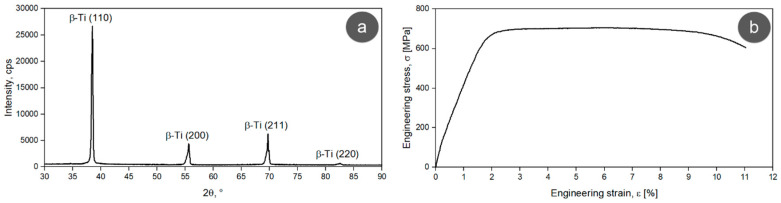
XRD spectrum (**a**) and stress–strain curve (**b**) of the AR TNZTSF alloy.

According to the XRD pattern presented in Figure 4a, the TNZTSF alloy in its as-received (AR) state exhibits a microstructure made up of a single β-Ti phase, which has the (110), (200), (211), and (220) corresponding planes. The β-Ti phase found in the composition of our studied alloy was indexed in an Im-3m body-centered cubic system, and its calculated lattice parameter was around a = 3.304(7) Å, while the mean of its lattice microstrain was around ε = 0.101(3)%.

The existence of a single β-Ti phase in the microstructure of our studied alloy can be justified by its chemical composition, knowing the fact that by adding β-isomorphous elements, like Niobium (29.72 wt.%) and Tantalum (4.97 wt.%), to produce Ti-based alloys, one will most likely obtain an alloy consisting of a single phase, thanks to their full solubility in β-Ti [36]. Although Zirconium (11.87 wt.%) is known to be a neutral element, its presence in Ti-based alloys, together with Nb and Ta, can slightly increase the β-Ti phase stability and enhance its strength [37,38,39].

The tensile strain–stress curve of the AR TNZTSF alloy is displayed in Figure 4b. As can be seen, the alloy went through an elastic deformation behavior, followed by a plastic deformation behavior prior to fracture. This is a typical curve for β-Ti alloys, showing, as a specific feature, the presence of a flat area while also having a single-yielding behavior. The flat area tells us that the stress values remain invariable when increasing the strain and that the ultimate strength and yield strength values are close to each other. What causes the alloy behavior during the deformation mechanism was studied on various types of Ti-based alloys, and it seems to be related mainly to the grain size and the β-phase fraction, which can cause stress-induced β → α″ martensitic transformation along with dislocations and deformation twins [40,41,42,43,44,45].

Based on this recorded tensile strain–stress curve, the mechanical characteristics expressed by the ultimate tensile strength (σ_UTS_), yield strength (σ_0.2_), fracture/failure strain (ε_f_), and elastic modulus (E) were calculated and are summarized in Table 2. Also, in Table 2, the microhardness value (HV0.1) of the TNZTSF alloy in its AR state, obtained from the microhardness test, can be found.

**Figure 5:**
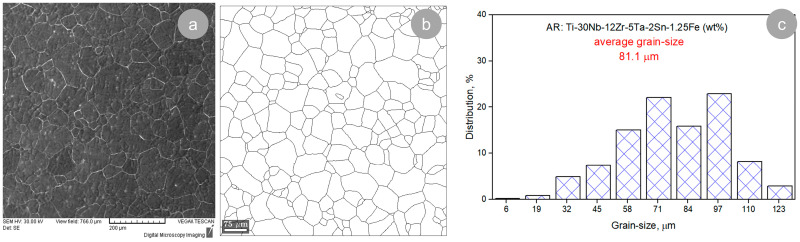
Typical microstructure image (**a**), grain morphology (**b**), and grain-size distribution (**c**) of the TNZTSF alloy in its AR state.

Figure 5a and 5b, respectively, present a typical microstructure image of the TNZTSF alloy in its AR state, showing a quite homogeneous microstructure and grains with a polyhedral equiaxed shape. As can be seen in Figure 5c, a large fraction of grains show grain sizes between 71 and 97 μm, while the average grain size of the AR TNZTSF alloy is situated close to 81.1 μm.

### 3.2. The Characterization of TNZTSF Alloy in the Cold-Deformed-by-Rolling (CR) State

**Figure 6:**
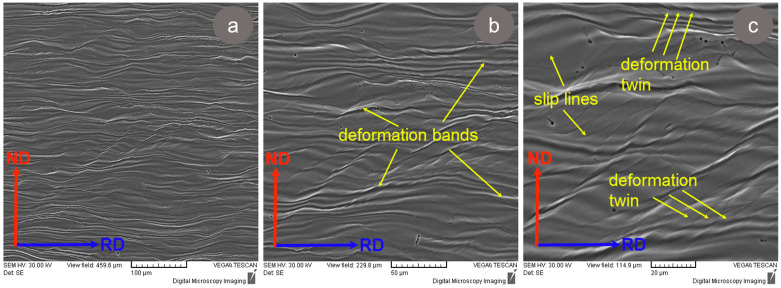
Typical microstructure images of the TNZTSF alloy in the cold-deformed-by-rolling (CR) state at ε = 60% at different magnifications: ×250 (**a**), ×500 (**b**), and ×1000 (**c**).

The microstructure of our TNZTSF alloy after cold deformation by rolling (CR) with a 60% deformation degree is presented in Figure 6. For investigating the progression of grain deformation, the samples were analyzed in the RD–ND cross-section (RD—rolling direction; ND—normal direction). In order to highlight the primary microstructural features, only the most representative images were chosen. 

In order to better showcase the microstructural features induced by the applied deformation degree, the SEM images were taken at different magnifications, such as ×250 (Figure 6a), ×500 (Figure 6b), and ×1000 (Figure 6c). As can be seen, after applying intense cold deformation, the polyhedral equiaxed grains became elongated along the RD processing direction, showing visible deformation bands and deformation twins.

Also, it can be remarked that the TNZTSF alloy subjected to cold deformation by rolling (CR) does not present discontinuities in volume, proving that the alloy’s microstructure remained homogeneous/undamaged even after applying intense cold deformation (60% deformation degree). Similar observations were also recorded in other studies performed on various types of Ti-based alloys [46,47,48].

It can also be seen that after cold deformation by rolling (CR), the β-Ti grains were strongly textured and that no other secondary phases, like ω, α′, or α″, were observed. Therefore, it can be concluded that the quantity of β-stabilizing elements found in the chemical composition of the TNZTSF alloy was enough to ensure a very good β-Ti phase stability and to suppress the formation of stress-induced secondary phases that could be formed during cold deformation.

**Figure 7:**
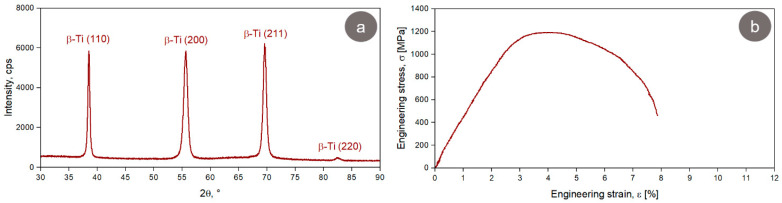
XRD spectrum (**a**) and stress–strain curve (**b**) of the TNZTSF alloy in the cold-deformed-by-rolling (CR) state at ε = 60%.

The XRD spectrum of the TNZTSF alloy in its CR state is presented in Figure 7a. One can observe the presence of a single β-Ti phase, the same one as in the case of the AR state (Figure 4a), having the same corresponding diffraction lines: (110), (200), (211), and (220). In this case, the calculated lattice parameter of the β-Ti phase was around a = 3.302(1) Å, and the mean of its lattice microstrain was around ε = 0.474(4)%. By comparing the β-Ti phase found in the AR TNZTSF alloy to the β-Ti phase found in the TNZTSF alloy in its CR state, it can be noticed that its calculated lattice parameter has a slight decrease, from a = 3.304(7) Å to a = 3.101(3) Å, which can be explained by the strain-hardening effect caused by intense cold-rolling deformation.

By analyzing the XRD spectra obtained before (AR state) and after cold-rolling deformation (CR state), it can be observed that the spectra exhibit some visible differences regarding diffraction line/peak width and intensity. The spectral peaks of the TNZTSF alloy in its CR state show a larger width in comparison with those of the TNZTSF alloy in its AR state, indicating an intense grain-refinement process as a result of the applied cold deformation. When analyzing the spectral peak intensities, one can observe an alignment of the (200) and (211) peaks with the (110) peak, indicating a crystallographic texturing effect, which is also caused by intense cold deformation. Similar observations were also reported in other studies [49,50,51].

Figure 7b shows a typical strain–stress curve obtained in the case of the TNZTSF alloy in its CR state, where it can be seen that the alloy subjected to a 60% deformation degree also exhibits a single-yielding behavior similar to the AR TNZTSF alloy’s case (Figure 4b). When comparing the AR with the CR state, it can be noticed that the TNZTSF alloy in its CR state shows limited plasticity compared to the TNZTSF alloy in the AR state. Also, one can notice that the TNZTSF alloy in the CR state exhibits a much higher ultimate tensile strength and yield strength compared to the TNZTSF alloy in its AR state, which can be attributed to the strain hardening caused by the increased dislocation and stress-induced deformation twin density.

The mechanical characteristics of the TNZTSF alloy in its CR state are given in Table 3, and by comparing them with those obtained for the TNZTSF alloy in the AR state (Table 2), it can be seen that the values of the ultimate strength (σ_UTS_) and yield strength (σ_0.2_) are clearly higher (ultimate tensile strength of 1192.1 MPa and yield strength of 1076.3 MPa, instead of ultimate tensile strength of 705.6 MPa and yield strength of 658.3 MPa obtained for the AR TNZTSF alloy). By analyzing the fracture/failure strain (ε_f_) and elastic modulus (E), one can notice that the values obtained after cold deformation by rolling decrease (fracture strain from 11.1% to 7.87% and elastic modulus from 55.6 GPa to 54.9 GPa). Table 3 also presents the microhardness of the CR state. One can notice that the microhardness value (HV0.1) is slightly higher, approx. 249 HV, in comparison with the AR state, approx. 226 HV.

When analyzing all mechanical properties for the AR and CR states presented in Table 2 and Table 3, respectively, one can conclude that the intense cold deformation induced a strong work-hardening effect in the TNZTSF alloy. Similar observations were reported in other studies concerning β-Ti alloys [52,53].

### 3.3. The Characterization of the TNZTSF Alloy in Its Solution-Treated (ST) State

In Figure 7, the XRD spectra of TNZTSF alloy specimens in the solution-treated (ST) state are illustrated. As observed, in all cases, only the diffraction lines/peaks belonging to the β-Ti phase were identified, namely, (110), (200), (211), and (220). Also, one can observe that the lattice parameter of the β-Ti phase shows a small increase, from 3.306(9) Å for a treatment duration of 2 min to 3.310(1) Å for a treatment duration of 30 min, indicating the occurrence of microstructural relaxation at the crystallographic level (see Table 4). In terms of the residual strain–stress field at the crystallographic level, the average microstrain shows a decrease from 0.611(2)% for a treatment duration of 2 min to 0.234(7)% for a treatment duration of 30 min, confirming the occurrence of microstructural relaxation at a crystallographic level as a direct consequence of the recrystallization phenomena in cold-deformed microstructures (see Table 4).

**Figure 8:**
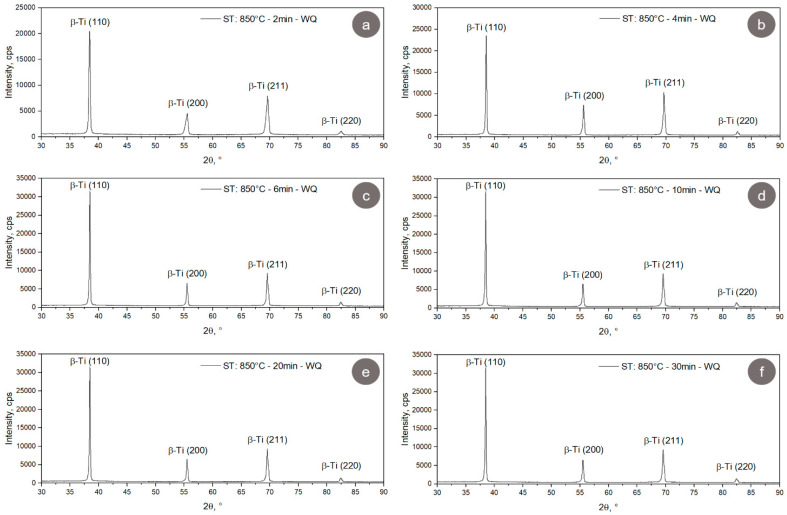
XRD spectra of the TNZTSF alloy in solution-treated (ST) states at 850 °C-2 min-WQ (**a**); 850 °C-4 min-WQ (**b**); 850 °C-6 min-WQ (**c**); 850 °C-10 min-WQ (**d**); 850 °C-20 min-WQ (**e**); 850 °C-30 min-WQ (**f**).

By comparing the CR state with the ST states in terms of diffraction line/peak intensity (see Figure 8), one can observe that the ST states show higher intensities when the solution treatment duration is increased, which suggests that the morphology and crystallinity of the recrystallized microstructures increase as the solution treatment duration increases. Also, when comparing the CR state with the ST states in terms of the diffraction line/peak width, one can observe that the ST states show smaller widths/sharper diffraction lines/peaks when the solution treatment duration increases, which suggests that the grain size of the recrystallized microstructures increases as the solution treatment duration increases.

**Figure 9:**
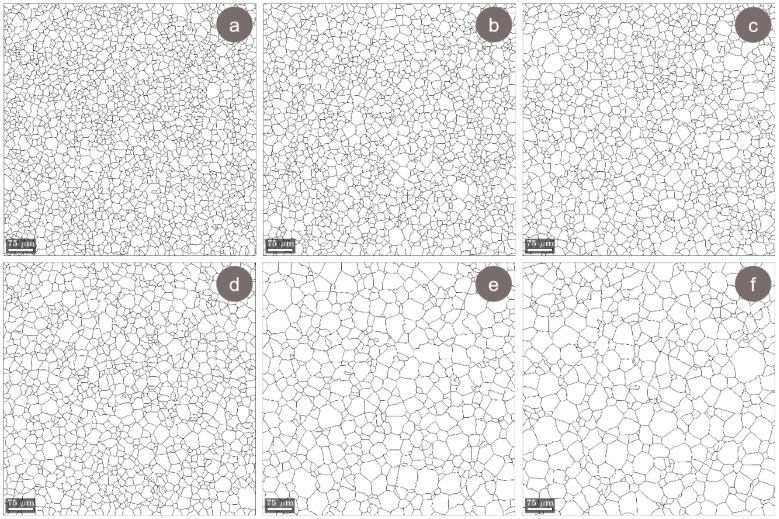
Typical grain morphology of the TNZTSF alloy in its solution-treated (ST) states at 850 °C-2 min-WQ (**a**); 850 °C-4 min-WQ (**b**); 850 °C-6 min-WQ (**c**); 850 °C-10 min-WQ (**d**); 850 °C-20 min-WQ (**e**); 850 °C-30 min-WQ (**f**).

**Figure 10:**
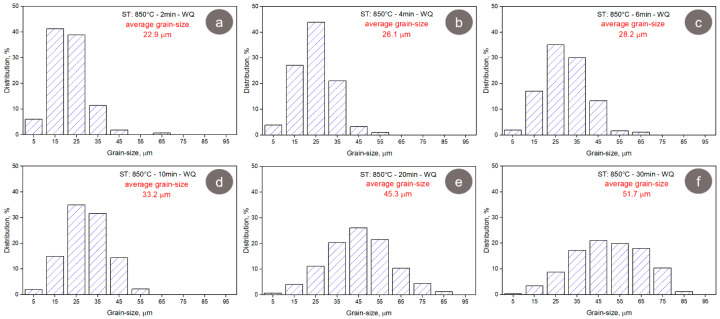
The distribution of the grain size in the TNZTSF alloy in its solution-treated (ST) states at 850 °C-2 min-WQ (**a**); 850 °C-4 min-WQ (**b**); 850 °C-6 min-WQ (**c**); 850 °C-10 min-WQ (**d**); 850 °C-20 min-WQ (**e**); 850 °C-30 min-WQ (**f**).

Figure 9 shows the grain morphology during the solution treatment (ST) of the CR TNZTSF alloy. As observed, the recrystallized microstructures show grains with polyhedral equiaxed morphology, which exhibits an increase in average grain size as the solution treatment duration increases. When analyzing the grain-size distribution and the average grain-size evolution, displayed in Figure 10, in all cases, a narrow grain-size distribution can be observed, with average grain sizes close to 22.9 μm for a treatment duration of 2 min, 26.1 μm for a treatment duration of 4 min, 28.2 μm for a treatment duration of 6 min, 33.2 μm for a treatment duration of 10 min, 45.3 μm for a treatment duration of 20 min, and 51.7 μm for a treatment duration of 30 min.

According to the Burke–Turnbull grain-growth evolution model, the grain-size evolution can be written as follows [54]:

(1) $$∆D^{2}=∆t\cdot K_{1}=∆t\cdot \left[2\cdot M\cdot \sigma \cdot C\right]$$

where *D* is the average grain size; ∆*t* is the duration of growth; *K*_1_ is the growth constant; *M* is the interfacial displacement constant; *σ* is the interfacial energy (the energy per unit area associated with an interface or surface, representing the energy expended in forming the contact area or grain boundary); and *C* is the growth parameter.

Starting from the Burke–Turnbull model, one can write/assimilate the treatment duration influence on the grain-size evolution using a linear equation:

(2) y=a+b·x

where $y=D^{2}$ is the grain size; *a* is the growth constant; and $b=\left[2\cdot M\cdot \sigma \cdot C\right]$ and represents the growth parameter.

**Figure 11:**
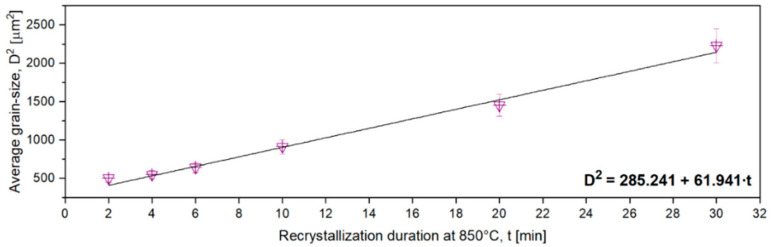
The variation in grain size during solution treatment (ST) at 850 °C, covering durations from 2 min to 30 min.

Figure 11 shows the grain-size evolution during solution treatment (ST) at 850 °C, with a treatment duration varying from 2 min to 30 min. By fitting, using linear regression, the obtained data, Equation (3) is obtained. As observed, linear regression accurately fits the experimental data. In the regression Equation (3), the growth constant shows a value close to a=285.241, while the growth parameter is close to b=61.941.

(3) $$D^{2}[\mu m^{2}]=285.241+61.941\cdot t[min]$$

In Table 5, the mechanical properties of the TNZTSF alloy in all solution-treated (ST) state durations are presented. Upon an initial examination of the data, it is noticeable that the strain-hardening effects caused by cold deformation are eliminated as a result of recrystallization phenomena.

By analyzing the strength characteristics (ultimate tensile strength and yield strength), one can observe that the strength properties show an increase for a treatment duration of up to 10 min, followed by a decrease. The ultimate tensile strength shows an initial value close to 906 MPa for a treatment duration of 2 min, 996 MPa for a treatment duration of 10 min, and finally, 946 MPa for a treatment duration of 30 min. Similar behavior is observed in the case of yield strength, with 855 MPa for a treatment duration of 2 min, 941 MPa for a treatment duration of 10 min, and finally, 903 MPa for a treatment duration of 30 min.

By examining the ductility characteristics (elongation to fracture/fracture strain), it can be noted that the fracture strain rises as the solution treatment duration increases, from 10.9% for a treatment duration of 2 min to 12.1% for a treatment duration of 4 min, followed by a constant decrease to 5.7% for a treatment duration of 30 min.

If the evolution of the elastic modulus during the solution treatment is analyzed, we can say that no significant variations are noted, with the average value of the elastic modulus being situated between 58.9 GPa for a treatment duration of 2 min and 54.8 GPa for a treatment duration of 30 min.

The analysis of microhardness evolution during solution treatment shows a continuous increase, from 228 HV0.1 for a treatment duration of 2 min to 381 HV0.1 for a treatment duration of 30 min. This continuous increase in microhardness must have occurred due to the thermally induced β-Ti → ω-Ti phase transformation, with it being known that nanometric ω-Ti-phase precipitates can be formed in the β-Ti grains during solution treatment as a consequence of the chemical inhomogeneity in β-Ti grains, especially Fe and Sn [55,56].

The remarks mentioned above about the microstructural and mechanical characteristics of the TNZTSF alloy in its solution-treated (ST) states collectively suggest that the phenomena occurring during solution treatment are primarily connected to the recrystallization of the cold-deformed microstructure. This process involves the regeneration of grains (dynamic Hall–Petch effect) and the elimination of the strain-hardening effect induced by cold deformation. This elimination is achieved by reducing the density of crystal defects and remanent strain–stress fields [57,58,59,60].

## 4. Conclusions

The studies carried out in this paper were focused on the impact of cold deformation by rolling and the solution treatment duration on the microstructural and mechanical characteristics of a Ti-30Nb-12Zr-5Ta-2Sn-1.25Fe wt.%) alloy. The key findings are summarized as follows:

- The manufacture of a β-type Ti-30Nb-12Zr-5Ta-2Sn-1.25Fe (wt.%) alloy, referred to as TNZTSF, was effectively achieved through the melting process in a cold-crucible induction furnace.
- The microstructure of the TNZTSF alloy in its as-received (AR) state reveals a uniform β-Ti phase featuring equiaxed polyhedral grains exhibiting a narrowly distributed grain size. The average grain size is approximately 81 μm.
- The cold-rolling (CR) process applied to the TNZTSF alloy, having a total deformation degree (total thickness reduction) of 60%, leads to a high dislocation density, together with high deformation twin and deformation band densities, indicating the occurrence of intense strain-hardening phenomena.
- Raising the solution treatment duration from 2 min to 30 min results in the formation of recrystallized polyhedral equiaxed grains of the β-Ti phase, following the grain-growth model proposed by Burke and Turnbull. These recrystallized grains exhibit an average size that varies from approximately 23 μm to 52 μm.
- The evolution of mechanical characteristics during the solution treatment process indicates that the strength properties continuously increase up to a treatment duration of 10 min, when a maximum value of 996 MPa is obtained in the case of ultimate tensile strength and a maximum value of 941 MPa is obtained in the case of yield strength, followed by a continuous decrease; the ductility is continuously increased up to a treatment duration of 4 min, when a maximum value of 12.1% is obtained in the case of fracture strain, followed by a continuous decrease, as a result of the recrystallization process in the cold-deformed-by-rolling microstructure, involving the regeneration of grains through the dynamic Hall–Petch effect and the elimination of strain hardening induced by cold deformation by rolling.
- The microhardness evolution during solution treatment shows a continuous increase up to 381 HV0.1 for a treatment duration of 30 min due to thermally induced nanometric ω-Ti-phase precipitates.
- An optimal mixture of mechanical characteristics, including high strength, superior ductility, and a low elasticity modulus, can be achieved through the application of cold deformation by rolling, followed by further solution treatment.

## Figures and Tables

**Table 1:** The quantitative chemical composition of the TNZTSF alloy in its as-received (AR) state.

Element	At. No.	Mass [wt.%]	Mass [at.%]	Abs. Error [%]	Rel. Error [%]
Titanium (Ti)	22	50.06	66.86	1.37	2.79
Niobium (Nb)	41	29.72	20.45	0.77	2.74
Zirconium (Zr)	40	11.87	8.32	0.27	2.85
Tantalum (Ta)	73	4.97	1.76	0.13	3.01
Tin (Sn)	50	1.98	1.01	0.07	3.91
Iron (Fe)	26	1.30	1.42	0.06	4.38
Sum		100.00	100.00		-

**Table 2:** Mechanical characteristics of the TNZTSF alloy in its AR state.

Structural State	Ultimate Strength, σ_UTS_ [MPa]	Yield Strength, σ_0.2_ [MPa]	Fracture Strain, ε_f_ [%]	Elastic Modulus, E [GPa]	Microhardness, HV0.1
AR TNZTSF alloy	705.6	658.3	11.1	55.6	226 ± 3

**Table 3:** Mechanical characteristics of the TNZTSF alloy in the CR state.

Structural State	Ultimate Strength, σ_UTS_ [MPa]	Yield Strength, σ_0.2_ [MPa]	Fracture Strain, ε_f_ [%]	Elastic Modulus, E [GPa]	Microhardness, HV0.1
CR TNZTSF alloy	1192.1	1076.3	7.87	54.9	249 ± 5

**Table 4:** Crystallographic parameter evolution of the ST TNZTSF alloy.

Crystallographic Parameters	Solution Treatment (ST): 850 °C-t-WQ					
	t = 2 min	t = 4 min	t = 6 min	t = 10 min	t = 20 min	t = 30 min
Lattice parameter, a [Å]	3.306(9)	3.306(1)	3.307(1)	3.307(9)	3.309(1)	3.310(1)
Average microstrain, ε [%]	0.611(2)	0.385(2)	0.347(1)	0.306(7)	0.240(7)	0.234(7)

**Table 5:** Mechanical property evolution of the TNZTSF alloy in its solution-treated ST states.

Structural State	Ultimate Strength, σ_UTS_ [MPa]	Yield Strength, σ_0.2_ [MPa]	Fracture Strain, ε_f_ [%]	Elastic Modulus, E [GPa]	Microhardness, HV0.1
ST: 850 °C-2 min-WQ	906	855	10.9	58.9	228
ST: 850 °C-4 min-WQ	946	887	12.1	55.6	236
ST: 850 °C-6 min-WQ	981	929	11.5	56.7	285
ST: 850 °C-10 min-WQ	996	941	8.2	56.9	342
ST: 850 °C-20 min-WQ	970	924	6.8	55.8	368
ST: 850 °C-30 min-WQ	946	903	5.7	54.8	381

## Data Availability

The data is contained within the article (due to).
